# Supplementary material for: Mapping the evolution of fertility support policies in China: A content and instrumental analysis
Source: PLoS One. 2025 Oct 9;20(10):e0332137. doi: 10.1371/journal.pone.0332137 (PMC12510515; doi:10.1371/journal.pone.0332137)
Supplement: S1 Appendix — (ZIP) [file pone.0332137.s001.zip › S1 Appendix. 226 original policy documents/26-国家卫生计生委办公厅关于做好生育登记服务工作的指导意见(FBM-CLI-4-270840).doc]

国家卫生计生委办公厅关于做好生育登记服务工作的指导意见

**发布部门：** [国家卫生和计划生育委员会(已撤销)](https://www.pkulaw.com/law/chl?Aggs.IssueDepartment=60319) [机构沿革](javascript:void(0))

**发文字号：**国卫办指导发〔2016〕20号

**发布日期：**2016.05.20

**实施日期：**2016.05.20

**时效性：** [失效](https://www.pkulaw.com/law/chl?Aggs.TimelinessDic=02)

**效力级别：** [部门规范性文件](https://www.pkulaw.com/law/chl?Aggs.EffectivenessDic=XE0302)

**法规类别：** [计划生育管理](https://www.pkulaw.com/law/chl?Aggs.Category=09705)

**失效依据：**[国家卫生健康委办公厅关于完善生育登记制度的指导意见](javascript:TMP(5112749,0,'chl'))

国家卫生计生委办公厅关于做好生育登记服务工作的指导意见
（国卫办指导发[2016]20号）

各省、自治区、直辖市卫生计生委，新疆生产建设兵团人口计生委：
　　实行生育登记服务制度，是实施全面两孩政策、改革完善计划生育服务管理的重要举措，是尊重家庭在计划生育中的主体地位、增强群众获得感和满意度的必然要求，是科学把握出生人口态势，精准提供妇幼健康、优生优育、计划生育服务的基础性工作。现就做好生育登记服务工作提出以下意见。
　　**一、**指导思想
　　以党的十八届五中全会精神为指导，深入贯彻《中共中央 国务院[关于实施全面两孩政策改革完善计划生育服务管理的决定](https://www.pkulaw.com/chl/8564756bae805261bdfb.html)》（中发〔2015〕40号）和《[中华人民共和国人口与计划生育法](https://www.pkulaw.com/chl/18c6c388fc14eaa9bdfb.html)》，坚持计划生育基本国策，本着以人为本、便捷高效、服务引领、信息支撑的原则，全面实行生育登记服务制度，引导群众负责任、有计划地生育，促进人口长期均衡发展。

　　**二、**登记对象和登记内容
　　（一）登记对象。夫妻生育第一个或第二个子女的，实行生育登记，由家庭自主安排生育。
　　夫妻应当在生育前进行生育登记；生育前未登记的，可在生育后及时补登。
　　（二）登记内容。生育登记的内容一般包括夫妻双方的婚姻信息、居住信息和现子女信息等。
　　登记时，夫妻双方需提供身份证或户口本、结婚证等有效证件。

　　**三、**登记机构和登记方式
　　（一）登记机构。县级卫生计生部门负责本区域生育登记服务的组织领导工作。
　　乡镇（街道）卫生计生机构具体办理生育登记服务工作。
　　村（居）民委员会卫生计生工作人员协助做好相关工作。
　　（二）登记方式。夫妻可在一方户籍地、现居住地的乡镇（街道）卫生计生机构办理生育登记。
　　无法亲自办理生育登记的，可委托村居（社区）卫生计生工作人员代为办理。
　　有条件的地方可以通过网上办事大厅、移动客户端等信息平台办理生育登记。

　　**四、**宣传倡导和服务保障
　　（一）宣传倡导。加强对做好生育登记服务工作的培训和宣传，提高卫生计生工作人员和广大群众对生育登记服务工作重要性的认识，明确生育登记程序、登记内容、登记方式和需要提供的材料、信息等，便民利民，维护群众合法权益。
　　（二）服务保障。卫生计生服务机构要及时为生育登记对象提供国家规定的基本公共卫生服务和计划生育基本技术服务，提高母婴健康水平。方便群众办理住院分娩补助、生育保险等手续。

　　**五、**工作要求
　　（一）各级卫生计生部门要高度重视做好生育登记服务工作，主要领导亲自抓，分管领导具体抓，制定具体办法，明确责任处室，加强工作统筹，确保任务落实。
　　（二）优化整合卫生计生服务资源，加强基层网络和队伍建设，充分发挥基层计划生育协会和计划生育专干的作用，落实必要工作经费，确保基层有人理事、有钱办事。
　　（三）大力推进生育登记、孕产期保健、住院分娩、出生医学证明、儿童预防接种等信息共享，运用信息化手段，优化工作流程，提高服务效率，确保信息安全。
　　（四）加强对生育登记服务制度落实情况的督导检查，及时总结推广好经验、好做法、好典型；对工作不力、敷衍塞责、弄虚作假的，要严肃追究责任。

国家卫生计生委办公厅
2016年5月20日

© 北大法宝：（[www.pkulaw.com](https://www.pkulaw.com/)) 专业提供法律信息、法学知识和法律软件领域各类解决方案。北大法宝为您提供丰富的参考资料，正式引用法规条文时请与标准文本核对。
欢迎查看所有[产品和服务](https://www.pkulaw.com/helps/2.html)。法宝快讯：[如何快速找到您需要的检索结果？](https://www.pkulaw.com/helps/70.html)[法宝](https://www.pkulaw.com/helps/69.html)V6有何新特色？

扫描二维码阅读原文

**原文链接：**<https://www.pkulaw.com/chl/db8e3faf7b8666d1bdfb.html>
